# The Extremely-Low-Frequency Electromagnetic Field Affects Apoptosis and Oxidative-Stress-Related Genes and Proteins in the Porcine Endometrium—An In Vitro Study

**DOI:** 10.3390/ijms25136931

**Published:** 2024-06-25

**Authors:** Pawel Jozef Wydorski, Agata Zmijewska, Anita Franczak

**Affiliations:** Department of Animal Anatomy and Physiology, Faculty of Biology and Biotechnology, University of Warmia and Mazury in Olsztyn, Oczapowskiego 1A, 10-719 Olsztyn, Poland; pawel.wydorski@uwm.edu.pl (P.J.W.); agata.zmijewska@uwm.edu.pl (A.Z.)

**Keywords:** extremely-low-frequency electromagnetic field, endometrium, in vitro, apoptosis, oxidative stress, peri-implantation, pig

## Abstract

Nowadays, the extremely-low-frequency electromagnetic field (ELF-EMF) is recognized as environmental pollution. The data indicate that the ELF-EMF may affect factors related to epigenetic regulation and alter important biological processes in the uterus. The impact of the ELF-EMF on apoptosis and oxidative-stress-related genes has not been documented in porcine endometrium. This raises the question of whether the exposure to the ELF-EMF can induce apoptosis and/or oxidative stress in the endometrium of pigs during the peri-implantation period. Porcine endometrial slices (100 ± 5 mg) collected (*n* = 5) during the peri-implantation period were treated in vitro with ELF-EMF at a frequency of 50 Hz and flux density of 8 × 10^4^ mG for 2 h. To determine the effect of ELF-EMF on apoptosis and oxidative stress in the endometrium, *CASP3*, *CASP7*, *CIDEB*, *GADD45G*, *NOS1*, *NOS2*, *NOS3*, and *TP53I3* mRNA transcript were analyzed using real-time PCR, and protein abundance of CASP3, CASP7 using Western blot, and eNOS using ELISA were determined. Moreover, CASP3/7 and NOS activity was analyzed using flow cytometry and colorimetry, respectively. The decreased CASP7 and increased NOS3 mRNA transcript and protein abundance in ELF-EMF-treated endometrium were observed. Moreover, *CIDEB*, *GADD45G*, and *TP53I3* mRNA transcript abundance was increased. Only *p* ≤ 0.05 was considered a statistically significant difference. The documented alterations indicate the potential of the ELF-EMF to affect apoptosis and generate oxidative stress in the endometrium. The insight into observed consequences documents for the first time the fact that the ELF-EMF may influence endometrial cell proliferation, angiogenesis, and/or tissue receptivity during peri-implantation.

## 1. Introduction

The extremely-low-frequency electromagnetic field (ELF-EMF) is non-ionizing electromagnetic radiation (NIR) [1], produced by everyday-used electronic devices such as electric blankets, induction hubs, or hair dryers, is defined as an external environmental pollutant [2,3,4,5]. The ELF-EMF is also used in physiotherapy and achieves satisfactory results in the treatment of joint pain, wound healing, or bone fractures [6]. Although it was concluded that the health impact of the ELF-EMF may be regarded as being relatively low [4], the ELF-EMF strength may be sufficient to affect biological functions [4,5]. It was demonstrated that the ELF-EMF induces changes in transcriptomic profile [7,8], DNA methylation level [9,10], the abundance of epigenetic-regulation-related factors [11], and steroidogenesis [12,13,14,15] in porcine endometrium and myometrium in vitro. Remarkably, it was also found that the ELF-EMF may evoke changes in apoptosis and oxidative stress in various mammalian cell types, i.e., human leukemia cell line K562, endothelial cells, HeLa cells, and mouse macrophages [16,17,18,19]. Interestingly, the biological systems may also interact with microwave radiation [20]. Nonetheless, it is especially interesting to investigate whether the exposure to the ELF-EMF at a frequency of 50 Hz can lead to induction of apoptosis and oxidative stress in the endometrium.

Apoptosis is a programmed cell death in which organisms eliminate unwanted cells or cells at the end of their life span and control intra-uterine homeostasis [21,22,23]. The process is controlled by the caspase family, among others [24,25]. Upon the receiving of the “death signal”, biochemical changes occur that lead to the activation of caspases and the initiation of proteolytic and nucleolytic processes in the cells, resulting in morphological changes and cell death [26]. Caspase 3 (and to a lesser extent caspase 7) plays the main role in the execution of apoptosis [27]. This enzyme is responsible for activating the caspase-activated-DNase (CAD) endonuclease, which in turn degrades DNA and causes chromatin condensation [28]. Caspase 3 is also involved in cell degradation and the formation of apoptotic bodies that are recognized by phagocytes [27]. Increased apoptosis of endometrial stromal cells at the site of implantation is important for the proper course of early pregnancy in pigs [29,30].

The obtained results indicated the involvement of several gene families, including the cell-death-inducing DFFA-like effector (CIDE) family [31,32] and cell-death-inducing DFFA-like effector beta (*CIDEB*) in inducing apoptosis [33]. Interestingly, it was also documented that the growth arrest and DNA-damage-inducible gamma gene (*GADD45G*) is involved in the regulation of cell growth or apoptosis induced by oxidative stress [34]. Apoptosis is closely related to the upregulation of GADD45 [35] and is also associated with the earlier onset of oxidative stress [36]. In most cell types, GADD45 proteins also cause cell cycle arrest [37].

Previously, it was found that the ELF-EMF alters *GADD45* mRNA transcript abundance in embryonic-stem-derived neural cells [38]. Taking into consideration that the ELF-EMF upregulates *GADD45G* in exposed endometrium [8], there is an interest in searching to understand whether the ELF-EMF causes oxidative stress in the endometrium. Oxidative stress can be defined as an imbalance between the production of reactive oxygen species (ROS) and the body’s antioxidant capabilities [39]. Nitric oxide (NO) is one of the oxygen-containing radicals produced by the enzymatic activity of nitric oxide synthase (NOS), encoded by *NOS1*, *NOS2,* and *NOS3* [40,41]. Three isoforms of NOS proteins have been identified, i.e., NOS I (neuronal NOS, nNOS), NOS II (induced NOS, iNOS), and NOS III (endothelial NOS, eNOS) [41]. In the mammalian endometrium, eNOS and iNOS have been localized in the glandular epithelium, the luminal epithelium, and the endothelial cells of the blood vessels in the non-gravid uterus [42,43,44,45]. The over-production of NO leads to the development of oxidative stress [46], and the detrimental effect of excess NO on endometrial receptivity and implantation has been demonstrated [47]. Increased expression of eNOS in the glandular epithelium and luminal endometrium has been determined in women with recurrent miscarriages and unexplained infertility [47].

There is also evidence that the ELF-EMF at a frequency of 50 Hz increases intracellular concentration of ROS in the K562 human leukemia cell line and HaCaT cells [16,48]. Increased ROS can cause DNA damage [49]. It was found that increased ROS production is correlated with the expression of tumor protein p53 inducible protein 3 (*TP53I3*, formally known as *PIG3*) [50,51]. The *TP53I3* encodes quinone oxidoreductase (QOR), which is involved in the DNA damage response and p53-mediated apoptosis [52]. When QOR binds the substrate, the ROS are produced in order for damaged cells to undergo apoptosis [53]. The ELF-EMF at a frequency of 50 Hz also has the potential to induce oxidative DNA damage in the blood plasma of Wistar rats after 50 and 100 days of exposition [54]. The question arises as to whether the ELF-EMF may trigger TP53I3-related apoptosis or oxidative stress in the endometrium during the peri-implantation period.

For ethical reasons, the effect of ELF-EMF treatment was investigated in a domestic pig model in vitro. There are similarities between humans and pigs in the chromosome structure and genome homology [55] and early embryonic development, including the prolonged conceptuses apposition and attachment phase [56,57]. Due to the physiological and anatomical similarities between human and pig, pigs are considered an animal model for research into early implantation in humans [58].

The peri-implantation period is extremely important for the success of pregnancy, and an appropriate molecular cross-talk between the embryo and the maternal uterine wall, as well as the proper intrauterine milieu, are necessary for the successful course of the implantation [59,60,61,62]. About 30% of embryos can be lost at the beginning of implantation [63,64]. Furthermore, uncontrollable apoptosis and ROS in the endometrium can be the reason for altered functions of the uterine tissues during the peri-implantation phase [65,66,67,68]. We hypothesized that the short time duration (two hours) of ELF-EMF treatment (50 Hz, 8 × 10^4^ mG) induces apoptosis and oxidative stress in the endometrium of pigs during the peri-implantation period. This exploratory hypothesis may generate more research concerning the impact of the ELF-EMF on biological systems. Specifically, the aim of this study was to determine whether ELF-EMF treatment in vitro impacts (1) the potential for apoptosis, determined by the abundance of *CIDEB*, *GADD45G* mRNA transcript, and CASP3, and CASP7 mRNA transcript, protein abundance, and the activity, and (2) oxidative stress generation, determined by the *NOS1*, *NOS2*, *NOS3*, and *TP53I3* mRNA transcript abundance and eNOS protein abundance and the NOS activity in the endometrium.

## 2. Results

### 2.1. The Relative mRNA Transcript Abundance of CASP3, CASP7, CIDEB, GADD45G, and TP53I3 in the Endometrium Exposed to ELF-EMF In Vitro

**Figure 1:**
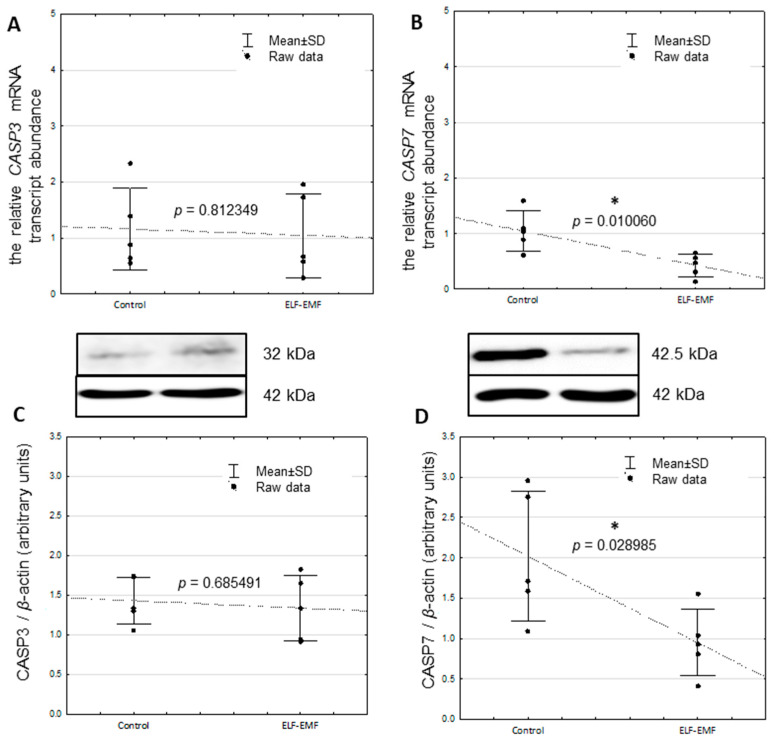
(**A**,**C**) CASP3 and (**B**,**D**) CASP7 mRNA transcript and protein abundance (32 kDa, 42.5 kDa, respectively) in control and ELF-EMF-treated (50 Hz, 2 h, 8 × 10^4^ mG) endometrial slices of pigs during the peri-implantation period (five biological replicates). Control and ELF-EMF-exposed slices were incubated separately in water-shaking baths. Data are presented as raw data ± SD (each data point in the figure consists of an average of two individual measurements). Student’s *t*-test was performed during the statistical analysis. In the protein abundance analyses, the mean value of the optical density of the proteins was normalized to the optical density of β-actin (42 kDa). For the analysis of CASP3, CASP7, and ACTB protein, separate 15% gels were prepared due to the molecular weights of the analyzed proteins being very similar. Asterisks above the bars imply statistically significant differences (* *p* < 0.05).

**Figure 2:**
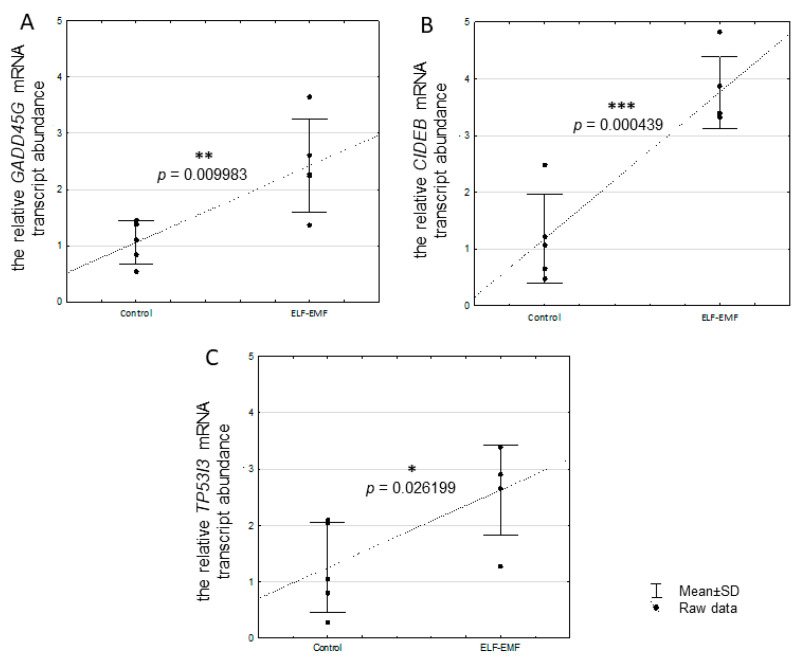
Relative abundance of *GADD45G* (**A**), *CIDEB* (**B**), and *TP53I3* (**C**) mRNA transcripts in the ELF-EMF-treated (50 Hz, 2 h, 8 × 10^4^ mG) endometrial slices collected from pigs (five biological replicates) during the peri-implantation period. Control and ELF-EMF-exposed slices were incubated separately in water-shaking baths. Data are presented as raw data ± SD (each data point in the figure consists of an average of two individual measurements). Student’s *t*-test was used for statistical analysis. Asterisks above the bars imply statistically significant differences (* *p* < 0.05, ** *p* < 0.01, *** *p* < 0.001).

There was no statistically significant difference in *CASP3* mRNA transcript abundance between control and ELF-EMF-treated tissue (Figure 1A, *p* = 0.812349). The decreased mRNA transcript abundance of *CASP7* was statistically significant in the ELF-EMF-treated endometrium (Figure 1B, *p* = 0.010060). In the endometrial slices exposed to ELF-EMF, *CIDEB* (*p* = 0.000439), *GADD45G* (*p* = 0.009983), and *TP53I3* (*p* = 0.026199) mRNA transcript abundance was statistically significant greater when compared to non-exposed (control) endometrial slices (Figure 2A–C).

### 2.2. The Relative Protein Abundance of CASP3 and CASP7 in the Endometrium Exposed to the ELF-EMF In Vitro

The CASP7 protein abundance was statistically significantly decreased in the ELF-EMF-treated endometrium compared to the control (Figure 1D, *p* = 0.028985). There was no statistically significant difference in the CASP3 protein abundance in the endometrium exposed to the ELF-EMF compared to the control (Figure 1C, *p* = 0.685491).

### 2.3. The In Vitro Effect of ELF-EMF on the Apoptosis Process in the Endometrium

**Figure 3:**
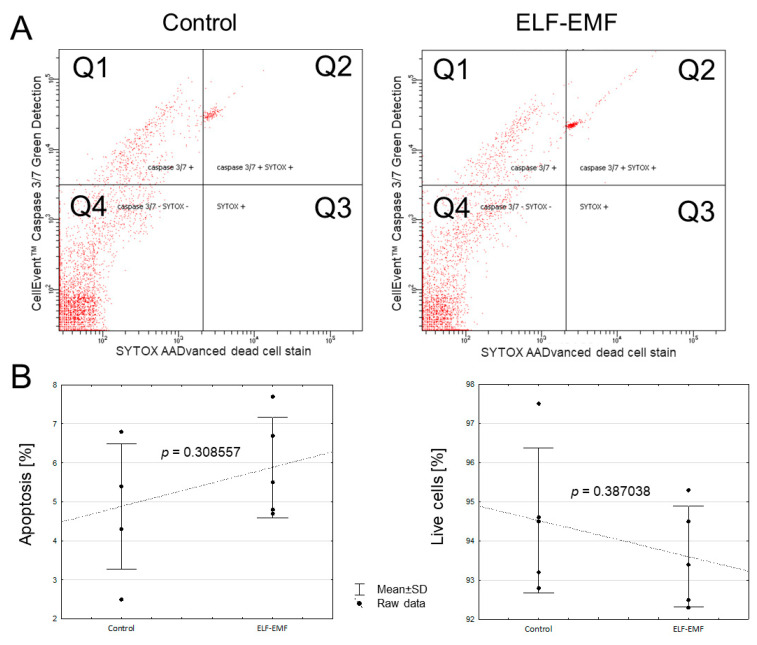
The assessment of activated CASP3 and CASP7 in cells isolated from endometrial slices treated in vitro with the ELF-EMF (50 Hz, 2 h, 8 × 10^4^ mG; the effect of the ELF-EMF on the apoptosis process). Endometrial slices were collected from pigs (five biological replicates) during the peri-implantation period. The control and the ELF-EMF-exposed slices were incubated separately in water-shaking baths. Cell apoptosis was evaluated using dual-color analysis (CellEvent™ Caspase-3/7 Green Detection Reagent and SYTOX AADvanced Dead Cell Stain) with a commercially available kit and the flow cytometry technique. Data are presented as scatter plots (**A**) and graphs illustrating the percent of apoptotic and live cells (raw data ± SD; (**B**)); each data point in the figure consists of an average of two individual measurements). Data obtained after flow cytometry analysis were analyzed by Student’s *t*-test. The quadrants of the scatter plot represent as follows: Q1 (upper left)—apoptotic cells, Q2 (upper right)—apoptotic and necrotic cells, Q3 (lower right)—necrotic cells, Q4 (lower left)—live cells.

Activity of CASP3 and CASP7 in the endometrium exposed to ELF-EMF was not altered compared to the control (Figure 3A, *p* = 0.751630)—There was no observed statistically significant effect of the ELF-EMF on the total percentage of cells in apoptosis (*p* = 0.308557), necrosis (*p* = 0.960981), and cell viability (*p* = 0.387038) (Figure 3B).

### 2.4. The Relative mRNA Transcript Abundance of NOS1, NOS2, and NOS3 in the Endometrium Exposed to the ELF-EMF In Vitro

**Figure 4:**
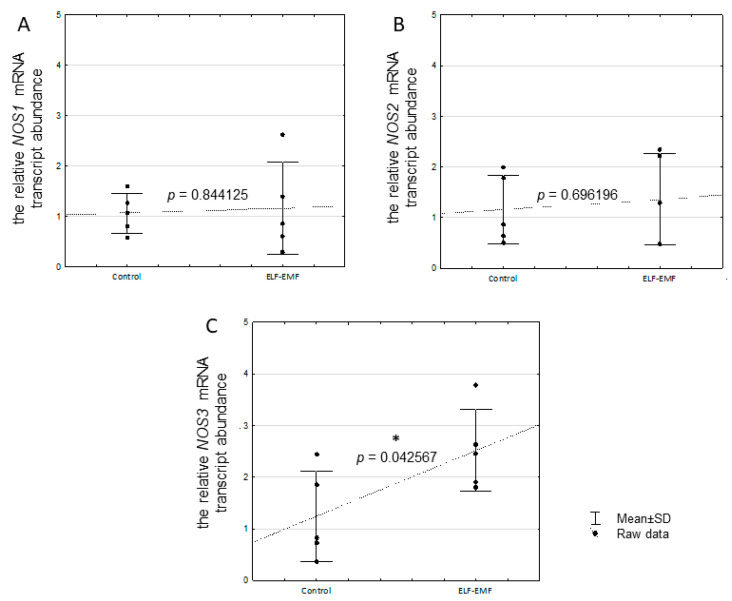
Relative abundance of *NOS1* (**A**), *NOS2* (**B**), and *NOS3* (**C**) mRNA transcripts in the ELF-EMF-treated (50 Hz, 2 h, 8 × 10^4^ mG) endometrial slices collected from pigs (five biological replicates) during the peri-implantation period. The control and the ELF-EMF-exposed slices were incubated separately in water-shaking baths. Data are presented as raw data ± SD (each data point in the figure consists of an average of two individual measurements). During the statistical analysis, Student’s *t*-test was performed. Asterisks above the bars imply statistically significant differences (* *p* < 0.05).

There was no statistically significant difference in the *NOS1* (*p* = 0.844125) and *NOS2* (*p* = 0.696196) mRNA transcript abundance in the endometrium exposed to the ELF-EMF (Figure 4A,B). The mRNA transcript abundance of *NOS3* (*p* = 0.04256) was statistically significantly increased in the ELF-EMF-treated endometrium compared to the control (Figure 4C).

### 2.5. The Abundance of eNOS Protein and NOS Activity (Nitrate + Nitrite Concentration) in the Endometrium Exposed to the ELF-EMF In Vitro

**Figure 5:**
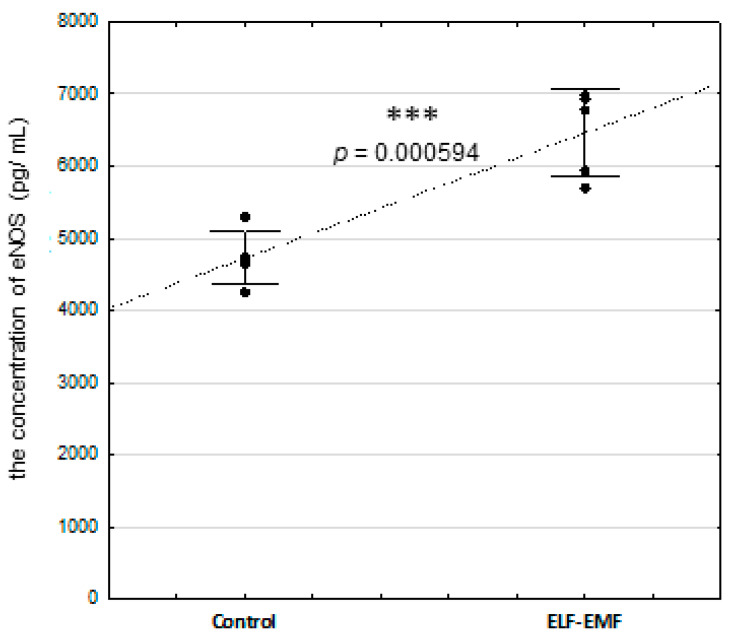
The concentration of eNOS in the ELF-EMF-treated (50 Hz, 2 h, 8 × 10^4^ mG) endometrial slices collected from pigs (five biological replicates) during the peri-implantation period. The control and the ELF-EMF-exposed slices were incubated separately in water-shaking baths. Data are presented as raw data ± SD (each data point in the figure consists of an average of two individual measurements). The statistical analysis was performed using Student’s *t*-test. Asterisks above the bars imply statistically significant differences (*** *p* < 0.001).

**Figure 6:**
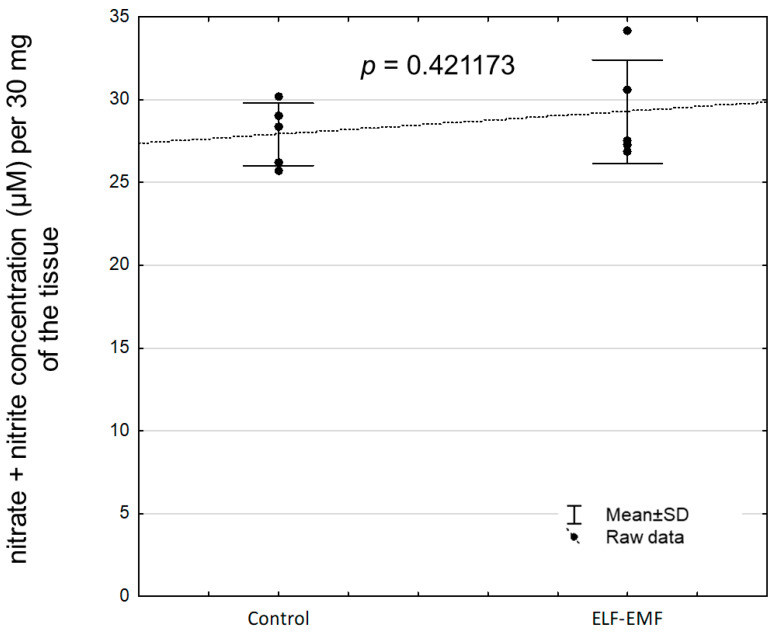
The nitrate and nitrite concentration in the ELF-EMF-treated (50 Hz, 2 h, 8 × 10^4^ mG) endometrial slices collected from pigs (five biological replicates) during the peri-implantation period. The control and the ELF-EMF-exposed slices were incubated separately in water-shaking baths. Data are presented as raw data ± SD (each data point in the figure consists of an average of two individual measurements). Statistical analysis was performed using Student’s *t*-test.

The concentration of eNOS protein was statistically significantly increased in the endometrium exposed to the ELF-EMF compared to non-exposed tissue (Figure 5, *p* = 0.000594). There was no statistically significant difference in nitrate and nitrite concentrations between the ELF-EMF-treated and control endometrium (Figure 6, *p* = 0.421173).

## 3. Discussion

The results of the present study documented that the short (two hours) duration of ELF-EMF treatment affects genes involved in apoptosis and oxidative stress generation in the endometrium collected during the peri-implantation period. The observed lowered abundance of CASP7 mRNA transcript and protein abundance may result in inhibition, while increased *CIDEB* and *GADD45G* mRNA transcript abundance may enhance the potential for apoptosis in the endometrium. Interestingly, increased NOS3 mRNA transcript and protein abundance, as well as increased *TP53I3* mRNA transcript abundance, may suggest enhanced potential for the generation of oxidative stress.

The caspases, in particular the executive CASP3 and CASP7, are responsible for carrying out apoptosis [24,25]. Remarkably, CASP7 has been found to have the function of detaching cells from the extracellular matrix, and in this case, it plays a role in the removal of apoptotic cells [69]. Decreased CASP7 mRNA transcript and protein abundance in the tissue exposed to the ELF-EMF indicates that, apart from a possible inhibition of apoptosis via ELF-EMF, endometrial cell detachment may also be impaired. In consequence, insufficient tissue structure or departure of damaged cells may occur.

Interestingly, the results of the current study demonstrated that the ELF-EMF reduced the CASP7/CASP3 ratio of mRNA (0.9:0.4) and protein (1.4:0.7). CASP3 and CASP7 play a key role in coordinating apoptosis, exhibiting almost indistinguishable activity [70,71], and they are independently functionally distinct [72]. Although reduction in the ratio of CASP7 to CASP3 level as a consequence of the ELF-EMF was documented, this reduction is not indicative of a beneficial or harmful effect on the endometrium. Future research should be provided to explain this phenomenon. Previously, it was found that *CASP7* knockdown results in the inhibition of HepG2 cell proliferation [73]. Therefore, this is additional evidence that the ELF-EMF may affect endometrial cell proliferation. In a previous study, we found that the ELF-EMF impacts the genes involved in epigenetic regulations. So, the epigenetic mechanisms may be involved in in apoptosis and oxidative stress generation. Decreased early growth response 2 (*EGR2*) mRNA transcript [8]; increased DNA methylation level of *EGR2* [9]; and decreased mRNA transcript of enhancer of zeste 2 polycomb repressive complex 2 subunit (*EZH2)*, embryonic ectoderm development (*EED*), and SUZ12 polycomb repressive complex 2 subunit (*SUZ12*), determined in endometrial tissue exposed to the ELF-EMF [11], support this thesis. Interestingly, reduced cell proliferation may be a sign of a cellular stress response [1]. Therefore, the current study provided evidence that the ELF-EMF potentially may be one of the reasons.

Here, we also found that the ELF-EMF increases endometrial mRNA transcript abundance of *TP53I3* included in the p53 signaling pathway. The TP53I3-encoded tumor protein p53-inducible protein is involved in reactive-oxygen-species-induced apoptosis and DNA damage response [53]. It is also worth mentioning that the upregulation of the TP53 protein was determined in endometrial epithelial cells in women with recurrent miscarriages [74]. Moreover, the *TP53I3* overexpression was determined in non-receptive bovine endometrium [75]. Interestingly, KEGG pathway analysis indicated an upregulated p53 signaling pathway in the endometrium exposed to the ELF-EMF during the peri-implantation period [8]. Therefore, affecting TP53I3, the member of the p53 signaling pathway, the ELF-EMF may be considered as altering endometrial receptivity during the peri-implantation period. More studies are needed to confirm this hypothesis.

The present study determined that ELF-EMF increased *CIDEB* mRNA transcript abundance in the endometrium. It is known that overexpression of *CIDEB* yields apoptosis [32]. Interestingly, the next-generation sequencing (NGS) analysis also provided evidence that *CIDEB* is overexpressed in the endometrium exposed to the ELF-EMF [8]. CIDEB takes also a role in regulating lipid metabolism, especially in lipid droplet growth, very-low-density lipoprotein lipidation [76,77], and cholesterol homeostasis [78]. It cannot be neglected that the upregulation of *CIDEB* in the endometrium exposed to the ELF-EMF may affect cholesterol metabolism in the tissue. Interestingly, in endometrial tissue exposed to the ELF-EMF, differentially expressed genes (DEGs) related to the KEGG pathway “cholesterol metabolism”, i.e., *CIDEB*, *LRP2*, *LDLR*, and *CYP7A1*, were indicated as mostly downregulated, except for *CIDEB* [8]. Since cholesterol serves as a substrate for steroid hormones [79,80], altered cholesterol metabolism may affect steroidogenesis in the endometrium exposed to the ELF-EMF. Interestingly, decreased production of testosterone [14] and 17-β-estradiol [15] in the endometrium exposed to the ELF-EMF was also documented. Thus, increased *CIDEB* mRNA transcript abundance, as a result of ELF-EMF, may have multidirectional consequences in the endometrium—it may induce apoptosis and affect cholesterol homeostasis. Further studies are required to investigate this assumption.

In the present study, we determined that in the endometrium exposed to the ELF-EMF, the *GADD45G*, which encodes the GADD45G protein, is upregulated. This observation confirms the results of the NGS analysis of the endometrium exposed to the ELF-EMF [8]. Interestingly, the GADD45 proteins occur in three isoforms, namely, α, β, and γ, which are involved in DNA replication, cell proliferation, and survival by responding to various environmental factors [81,82]. It was found that a greater amount of GADD45 proteins leads to cell cycle arrest after DNA damage [83]. Furthermore, GADD45G is defined as proapoptotic and a growth-arrest protein [82]. Hence, a higher level of *GADD45G* mRNA transcript in the endometrium when exposed to the ELF-EMF might interfere with the cell cycle, leading to decreased cell proliferation and heightened apoptosis.

We reported here that the NOS3 mRNA transcript and protein abundance are increased in the endometrial tissue exposed to the ELF-EMF. The upregulation of *NOS3* in the porcine endometrium treated with the ELF-EMF was also demonstrated [8]. These results may indicate the possibility of oxidative stress generation by the ELF-EMF. However, in the present study, we also found that the NOS activity was not altered in the ELF-EMF-treated endometrium. Interestingly, the activation of eNOS was documented after a long duration of ELF-EMF treatment [84,85,86,87]. The eNOS activation was found after a 20 min exposition of human immortalized microvascular endothelial cells to the ELF-EMF at a frequency of 60 Hz for four days [84]. Moreover, the exposure of mice to the ELF-EMF at a frequency of 60 Hz for 48 h caused Ca^2+^-dependent eNOS activation and induced hyperalgesia with the increased NO synthesis in the brain and spinal cord [85]. Furthermore, the upregulation of eNOS and NO production was found in the human keratinocyte cell line HaCaT exposed to ELF-EMF at a frequency of 50 Hz for 18 h [86]. It was also found that in Rat-1 fibroblasts exposed to the ELF-EMF at a frequency of 50 Hz for 24 h, the increased content of ROS led to oxidative stress, and such a state persisted also after 24 h of exposure [87]. Therefore, the generation of oxidative stress in the consequences of the ELF-EMF may be related to the duration of ELF-EMF treatment.

Observed in the current study, increased eNOS protein abundance in endometrial tissue exposed to the ELF-EMF is an interesting issue. As mentioned, eNOS has been localized in the glandular epithelium, the luminal epithelium, and the endothelial cells of blood vessels of non-gravid uterus [42,43,44]. The consequences of induced alterations in eNOS expression may modulate angiogenesis. It was documented that the overabundance of the eNOS protein affects angiogenesis in the response to tissue ischemia in mice [88]. In addition, increased expression of eNOS was also found in the endometrial glandular and luminal epithelium of women with recurrent miscarriages and unexplained infertility [47]. Another study documented that a long (12 h) duration of ELF-EMF treatment at a frequency of 50 Hz induces angiogenesis in human endothelial cells in vitro [89]. Therefore, the increased NOS3 mRNA transcript and protein abundance in the endometrium exposed to the ELF-EMF could therefore indicate that a short duration of ELF-EMF treatment may induce angiogenesis in the endometrium. Future studies should determine in detail how this effect of the ELF-EMF at a frequency of 50 Hz interferes with angiogenesis in the endometrium during implantation.

To determine whether the ELF-EMF may induce oxidative stress in the endometrium, the concentration of NO metabolites in tissue homogenate, i.e., nitrate and nitrite, were measured. The NO concentration is routinely determined indirectly by measuring the nitrate and nitrite concentration in biological samples to measure eNOS activity [90]. Surprisingly, we found the lack of changes in total nitrate and nitrite concentration in the ELF-EMF-exposed endometrium. This notion suggests a lack of eNOS activity. Although the effect of the ELF-EMF on eNOS activity (expressed as altered NO metabolite concentration) was not visible, the eNOS mRNA transcript and protein abundance were increased. Interestingly, the results of the past study documented that increased production of NO may be accompanied by increased calcium ion concentration [91]. It cannot be excluded that the lack of eNOS activity could be the result of impaired calcium ions concentration and/or distribution in the tissue. In the ELF-EMF-exposed endometrium, downregulation of several genes closely associated with calcium homeostasis and distribution, i.e., the transient receptor potential cation channel subfamily M member 6 (*TRPM6*) and the transient receptor potential cation channel subfamily V member 1 (*TRPV1*) included in the transient receptor potential (TRP) family as well as the calcium voltage-gated channel auxiliary subunit alpha 2 delta 3 (*CACNA2D3*), were documented [8,92,93,94]. Interestingly, it was determined that the TRP family is sensitive to external mechanical or electrical signal stimuli [95]. It has been proposed that the TRP forms the bridge between the ELF-EMF and the changes in intracellular calcium ion concentration, paving the way for ELF-EMF-induced calcium oscillations [96]. In addition, KEGG pathway analysis showed downregulated “inflammatory mediator regulation of TRP channels” and “calcium signaling pathway” in the endometrium exposed to the ELF-EMF [8]. Importantly, calcium is an abundant intracellular messenger involved in the signaling pathways of numerous biological processes, including cell adaptation, survival, and death [97]. Ca^2+^ plays also a crucial role in decidualization and implantation processes [98]. Therefore, the ELF-EMF may affect implantation through the impact on insufficient calcium concentration and distribution.

It is worth noting that the ELF-EMF at a frequency of 50 Hz may play a role in oxidative damage via two intracellular pathways, namely, excessive MDA production and the Fenton pathway [99,100]. Induction of these pathways subsequently leads to biomolecular damage, such as DNA double-strand breaks, DNA/RNA and proteins damage, lipid peroxidation leading to a range of systemic disorders, or cell death [3,54,101]. Surprisingly, the ELF-EMF may result in reduced antioxidant capacity in blood samples from people who constantly use hair dryers in hairdressing salons [102]. Due to the fact that the above-mentioned changes were mainly observed after long duration of ELF-EMF treatment, future studies should determine how a longer duration of ELF-EMF treatment may affect oxidative stress in the pig endometrium during the peri-implantation period. The question arises as to why the ELF-EMF at a frequency of 50 Hz causes cell proliferation, angiogenesis, and endometrial receptivity. It is noteworthy that alterations in DNA methylation level, as well as altered expression of genes related to apoptosis, angiogenesis, and endometrial receptivity, were documented in the endometrium exposed to the ELF-EMF [9,11]. Epigenetic mechanisms may be related to the observed changes. So far, the direct mechanisms through which the ELF-EMF causes biological processes has not been determined. Thorough examination of the potential underlying mechanisms behind the observed effects should be performed in the future.

## 4. Materials and Methods

### 4.1. Ethics

The approval of an Ethics Committee is not required when in vitro research is based on tissues of farm animals obtained during regular economic slaughter, and it is not formerly assumed by any procedure. All biological tissues used in the present research were collected during economic slaughter by qualified personnel. The pigs were not handled by the authors. The agreement of the ethical committee is not needed according to the legislation provided by the European directive (2010/63/EU of the European Parliament and of the Council—Article 3, Paragraph 1) nor in the Polish Act—“Act on the Protection of Animals Used for Scientific or Educational Purposes” of 15 January 2015 (Journal of Laws of 2015 Item 266; Article 2, Paragraph 1, Point 6).

### 4.2. Animals, Endometrial Slice Collection, and ELF-EMF Exposure

Uteri were collected post-mortem from the pubertal pigs (*n* = 5, *Sus scrofa domestica* L., Polish Landrace × Polish Great White, 90–110 kg) during the peri-implantation period (15–16 days of pregnancy). The tissues were immediately transported to the Laboratory of the Department of Animal Anatomy and Physiology, Faculty of Biology and Biotechnology, University of Warmia and Mazury in Olsztyn, Poland. The experimental design is shown in Supplementary Figures S1 and S2. Before starting the experiment, the ELF-EMF parameters were tested by a member of Astar’s professional service and support team. The ELF-EMF distributions were determined by an Astar employee prior to the experiment: GM04 magnetic field meter (Hirst Magnetic Instruments, Falmouth, UK), Hall effect sensor type A1321 (Allegro MicroSystems, Manchester, NH, USA), TDS1002B oscilloscope (Tek-tronix, Beaverton, OR, USA), and BM515X digital multimeter (BRYMEN, New Taipei, Taiwan). The endometrial slices (100 mg ± 5 mg; 2–3 mm thick, five biological repeats, *n* = 5) from the middle part of the uterine horns were prepared using sterile scissors and forceps as previously described [8,9,11]. In the next step, endometrial slices were preincubated in a water-shaking bath at 37 °C in vitro for two hours in the two separate 24-well culture plates, in which wells were filled with 1 mL or 2 mL of medium 199 (Sigma Aldrich, St. Louis, MI, USA). After preincubation, medium 199 was rechanged to fresh 1 mL or 2 mL of medium. The slices were divided into the control group (non-exposed to ELF-EMF, Sham’s conditions) or a group exposed to ELF-EMF. There was no time delay between the processing of the control and exposed samples. Endometrial slices (non-exposed and exposed to ELF-EMF) were intended for mRNA and protein analysis (one endometrial slice with 1 mL of medium 199 in the well; three technical repeats, *n* = 5), and for analysis, we used a flow cytometer (two endometrial slices with 2 mL of medium 199 in the well; three technical repeats, *n* = 5). The endometrial slices were exposed for two hours to constant, sinusoidal ELF-EMF at a frequency of 50 Hz and magnetic field of 8 × 10^4^ mG using the ELF-EMF generator (Magneris device—Astar; https://www.astar.pl/produkty/magneris; accessed on 20 May 2017) as described previously [8,9,12,103]. The generator was equipped with flat applicators, by which sinusoidal, triangular, or rectangular waveforms of ELF-EMF can be emitted with frequencies ranging from 2 to 120 Hz [104]. Control plates with endometrial slices were incubated separately in the water-shaking bath to prevent ELF-EMF exposition. After incubation, endometrial slices (with the exception of slices intended for flow cytometer analysis) were collected and frozen in liquid nitrogen (−196 °C), and then they were placed in an ultra-freezer (−80 °C) until future analysis.

### 4.3. RNA Isolation

The isolation of total RNA from fragments of endometrial slices (≈30 mg) non-exposed (*n* = 5) and exposed to ELF-EMF (*n* = 5) was started by adding 500 µL of Extrazol (Blirt, Gdańsk, Poland) and RNeasy Mini Kit (Qiagen, Germantown, TN, USA), according to the protocol previously described [11,13]. Afterwards, tissues were homogenized three times for 10 s in a diethyl-pyrocarbonate-treated Eppendorf tube. Then, the samples were incubated on ice for 30 min, mixing once every 10 min. In the next step, to extract RNA from homogenates, freezing chloroform was added, and samples were centrifuged at 8000× *g* for 8 min at 4 °C. The top layer obtained for each sample was collected to the new Eppendorf tube, and then ice-cold 70% ethanol (−20 °C) was added to purify the RNA. All of the mixture was put into the RNeasy Mini spin column according to the protocol (Qiagen, USA), and the next steps of RNA isolation were performed according to the manufacturer’s procedure. At the end, RNA was eluted with 30 μL grade water (Ambion, Thermo Fisher Scientific, Waltham, MA, USA). The quality and quantity of the eluted RNA was checked spectrophotometrically with a spectrophotometer, namely, Infinite M200 Pro (Tecan, Männedorf, Switzerland). All samples were then stored at −80 °C until quantity real-time PCR (qRT-PCR) was performed.

### 4.4. Determination of Relative mRNA Transcript Abundance of CASP3, CASP7, CIDEB, GADD45G, NOS1, NOS2, NOS3, and TP53I3

The endometrial *CASP3*, *CASP7*, *CIDEB*, *GADD45G*, *NOS1*, *NOS2*, *NOS3*, and *TP53I3,* and two reference genes, i.e., β-actin (*ACTB*) and glyceraldehyde-3-phosphate dehydrogenase (*GAPDH*), relative mRNA transcript abundance were determined with one-step qRT-PCR using the TaqMan RNA-to-Ct 1-Step Kit (Applied Biosystems, Foster City, CA, USA) and the commercially available TaqMan probes presented in Table 1 (Thermo Fisher Scientific, USA).

The total reaction volume was 10 μL, in which 4 pg/μL of total RNA for *CIDEB*, *TP53I3*, *NOS1*, *NOS2*, *NOS3*, *CASP3*, *CASP7*, *ACTB,* and *GAPDH* or 16 pg/μL of total RNA for *GADD45G*, *ACTB,* and *GAPDH* (the expression of *ACTB* and *GAPDH* obtained for 16 pg/μL was only used for the analysis of *GADD45G* mRNA transcript abundance) were added. The parameters of the reaction were as follows: one cycle of reverse transcription at 48 °C for 15 min, followed by one hot start step at 95 °C for 10 min, and 40 cycles for *GADD45G,* as well as 50 cycles for *NOS1*, *NOS2*, or *NOS3* or 60 cycles for *CASP3*, *CASP7*, *TP53I3*, *CIDEB*, *ACTB,* and *GAPDH* of amplification at 95 °C for 15 s, and 60 °C for 1 min. The ΔΔCt method [105] was used to calculate the relative mRNA transcript abundance with two reference genes.

### 4.5. Protein Isolation

Total proteins were isolated from endometrial slices (≈30 mg) according to the previously described protocol [10,11], where the isolation buffer contained the following: T-Per (Thermo Fisher Scientific, USA) supplemented with (10 μL/500 μL) Halt protease inhibitors (Thermo Fisher Scientific, USA). In brief, 500 μL of isolation buffer was added to Eppendorf tubes containing the harvested fragments of endometrial slices. The tissues were then homogenized and placed on ice for 30 min (shaking three times every 10 min). After incubation, the samples were centrifuged three times at 10,000× *g* for 10 min until the samples were clarified. The supernatants were then transferred to new Eppendorf tubes, and the extracted protein samples were placed in the ultra-freezer (−80 °C) overnight. The next day, the Bradford method was used to measure the protein concentration. Absorbance was evaluated at 595 nm with TECAN M200 Pro microplate reader and Magellan software (version 7.2, Tecan, Switzerland).

### 4.6. Determination of Pro-CASP3 and Pro-CASP7 Protein Abundance

The abundance of pro-CASP3 (32 kDa), pro-CASP7 (42.5 kDa), and ACTB (42 kDa) proteins was determined by Western blot analysis according to the previously described protocol [10,11]. The 30 μg for pro-CASP3 and 40 μg for pro-CASP7 aliquots of total protein were adjusted to a total volume of 10 μL and combined with 10 μL of 6 × Laemmli sample buffer and DTT (1:1). Next, samples were denatured at 99 °C for 3 min. Samples were lined up in a 4% stacking gel (85 V/gel and 15 mA/gel) in the first step of electrophoresis and then resolved in a 15% running gel (100 V/gel and 19 mA/gel for pro-CASP7 and ACTB or 110 V/gel and 22.5 mA/gel for pro-CASP3 and ACTB) in 1 × Tris-glycine-SDS buffer. For the analysis of the ACTB protein, separate 15% gels were prepared due to the molecular weights of the analyzed proteins being very similar. The separated proteins were transferred to PVDF membranes (0.2 µm) in the next step. Wet electro-transfer (85 V, 204 mA, 20 W, and 1 h) was used for pro-CASP3 and ACTB, and semi-dry electro-transfer (25 V, 2.4 mA, and 12 min) was used for pro-CASP7 and ACTB. The membranes were then blocked in 5% bovine serum albumin fraction V in TBST buffer for 1 h at room temperature (RT). After blocking, the membranes were incubated overnight at 4 °C with the primary antibodies specified in Table 2.

The next day, blots were placed in the secondary HRP-conjugated goat anti-mouse antibodies for pro-CASP3 and pro-CASP7 or goat anti-rabbit antibodies for ACTB (Table 2) at RT. Immobilon Western Chemiluminescent HRP Substrate staining solution was used for the visualization of bands (Merck, Darmstadt, Germany) during a 5 min incubation period and was visualized in Azure Biosystems. The optical density (OD) was measured using ImageJ (version 1.52a, open-source software). The OD of the ACTB bands was used to normalize and calculate the abundance of the pro-CASP3 and pro-CASP7 proteins.

### 4.7. Endometrial Cell Isolation and Flow Cytometer Analysis of CASP3/7 Activity

To measure ELF-EMF-induced CASP3/7 activity, the endometrial cell isolation after two hours of in vitro incubation (in the presence or absence of ELF-EMF exposure) was performed. To the ELF-EMF-treated and non-treated endometrial slices (6 slices for one biological replicate, ≈600 mg), 6 mL of early prepared warm 0.2% collagenase from Clostridium histolyticum (C9263, Merck, Germany) in Hanks′ Balanced Salt solution was added. The digestion of the tissues was in a water bath at 37 °C for 30 min. After digestion, the suspended cells were centrifuged at 1000× *g* for five minutes. The supernatant was removed and 200 µL of medium 199 was added to wash the cells by pipetting. The cells were then centrifuged again (1000× *g*, 5 min), and the supernatant was poured off. The cells were filtrated and next resuspended in 6 mL of warm (37 °C) phosphate-buffered saline (PBS, pH = 7.4).

Endometrial CASP3/7 activity was estimated by two-color cytometer analysis using CellEvent^TM^ Caspase-3/7 Green Detection Reagent (ThermoFisher, Carlsbad, CA, USA) according to the manufacturer’s protocol. The green-fluorescent CellEvent™ is a fluorogenic substrate that consists of a four-amino-acid peptide (DEVD) conjugated to a nucleic acid binding dye and is used for the detection of activated CASP3 and CASP7. Active CASP3 and CASP7 are able to cleave the caspase3/7 recognition sequence encoded in the DEVD peptide. Binding of DNA and cleavage of the recognition sequence by the CellEvent^TM^ reagent labels cells in the apoptosis phase, while the red-fluorescent SYTOX™ AADvanced™ reagent was used to easily discriminate apoptotic cells from live and necrotic cells. The positive control for apoptosis was prepared using cells treated with high temperatures (99 °C) for 5 min. For the analyses, the cells were diluted in PBS (0.5 mL of the previously suspended cells and 0.5 mL PBS). Measurements were performed using a FACSCelesta™ flow cytometer (BD Biosciences, Fremont, CA, USA). Data were obtained and analyzed using FACSDiva 9.0 software (BD Biosciences, Franklin Lakes, NJ, USA). For data acquisition and results analysis, a total of 30,000 events were collected from each sample during flow cytometry analysis.

### 4.8. Measurement of NOS Activity In Vitro—Nitrate/Nitrite Colorimetric Assay (LDH Method)

The measurement of nitrate and nitrite concentrations was performed with the commercial colorimetric Nitrate/Nitrite Assay Kit (760871, Cayman Chemical, Ann Arbor, MI, USA), according to the manufacturer’s procedure. Nitrite (NO_2_^−^) and nitrate (NO_3_^−^) are known as inert oxidative end products of endogenous NO metabolism [106]. The analysis was conducted according to the manufacturer’s protocol for tissue homogenate. To prepare the homogenates, ≈30 mg of endometrial tissue/each biological repeat/each studied group was performed. The samples were homogenized in 500 μL of PBS (pH 7.4), and for measurement, 40 µL of prepared samples was used. Every sample was measured in duplicate. The absorbance was read at 540 nm using a plate reader Infinite M200 Pro with i-control™ software (version 1.11, Tecan, Switzerland). The nitrate and nitrite concentrations were interpolated from the standard curve. The fit of the standard curve was confirmed by the coefficient of determination (R^2^ = 0.996).

### 4.9. The eNOS Concentration

The concentration of endometrial eNOS was determined with the specific for the porcine eNOS commercial sandwich ELISA (ELK5617, ELK Biotechnology, Denver, CO, USA) according to the manufacturer’s protocol. The detection range of this kit is 15.63–1000 pg/mL, and the sensitivity is 6.1 pg/mL. According to the manufacturer, no significant cross-reactivity or interference between pig eNOS and the analogs was observed. The tissue homogenates prepared for the measurement of NOS activity were also used to measure the eNOS concentration. The most effective dilution of the tissue homogenate was determined by a preliminary test. Therefore, tissue homogenates were fivefold diluted. Absorbance was measured at 450 nm using an Infinite M200 Pro plate reader with i-control™ software (version 1.11, Tecan, Switzerland). All samples were analyzed in two technical repeats. The porcine eNOS concentrations in the samples were interpolated from the standard curve. The fit of the standard curve was confirmed by the coefficient of determination (R^2^ = 0.9756).

### 4.10. Statistical Analysis

All results are presented as raw data ± standard deviation (SD) of experiments for all biological replicates (*n* = 5). Only *p*-values equal to or less than 0.05 were considered statistically significant differences. The 2^−ΔΔCt^ values were used for the analysis of mRNA transcript abundance. The mean values of OD of protein bands were used for the determination of studied protein abundance. For the analysis of CASP3/7 activity, the means of the percent of cells were used. For the analysis of NOS activity, the concentration of nitrate and nitrite was used. For the analysis of eNOS protein abundance, the concentration of eNOS was used. The normal distribution of the results obtained was checked using the Shapiro–Wilk test, and all results were normally distributed. The significance of all data was analyzed by Student’s *t*-test using Statistica software (version 13.3, StatSoft, Tulsa, OK, USA).

## 5. Conclusions

The results of the present study indicate that the short period of the ELF-EMF treatment (two hours) at a frequency of 50 Hz possesses the potential to affect apoptosis-related factors and may induce oxidative stress in the endometrium of pigs during the peri-implantation period. The observed changes induced by the ELF-EMF suggest that the ELF-EMF may have an impact on cell proliferation, angiogenesis, and endometrial receptivity. Future studies should investigate how the long duration of ELF-EMF treatment affects biological processes in the uterus such as proliferation, angiogenesis, apoptosis, and oxidative stress formation.

## Figures and Tables

**Table 1:** TaqMan probes used in the mRNA transcript abundance analyses using RT-PCR.

TaqMan Probes	Target Gene Name	Assay ID
*CASP3*	Caspase 3	Ss03382792_u1
*CASP7*	Caspase 7	Ss06867774_m1
*CIDEB*	Cell-death-inducing DFFA-like effector B	Ss03389752_g1
*GADD45G*	Growth arrest, and DNA damage inducible 45γ	Ss04246840_g1
*NOS1*	Nitric oxide synthase 1	Ss06838170_m1
*NOS2*	Nitric oxide synthase 2	Ss03374608_u1
*NOS3*	Nitric oxide synthase 3	Ss03383840_u1
*TP53I3*	Tumor protein P53 inducible protein 3	Ss06911275_g1
Reference genes		
*ACTB*	β-actin	Ss03376081_u1
*GAPDH*	Glyceraldehyde-3-phosphate dehydrogenase	Ss03374854_g1

**Table 2:** The primary and secondary antibodies used for Western blot analyses.

Primary Antibody	Catalog Number	Dilution	Company	Host
CASP3	MA1-91637	1:1000	Invitrogen	Mouse
CASP7	NBP1-19229	1:1000	Biotechne	Mouse
Secondary antibody				
Anti-rabbit HRP	314460	1:5000	Invitrogen	Goat
Anti-mouse HRP	31430	1:5000	Invitrogen	Goat
Reference protein				
ACTB	A2066	1:200	Sigma Aldrich	Rabbit

## Data Availability

All of the data are presented in the study.

## Supplementary Materials

The following supporting information can be downloaded at https://www.mdpi.com/article/10.3390/ijms25136931/s1.
