# Supplementary figures and images for: The Extremely-Low-Frequency Electromagnetic Field Affects Apoptosis and Oxidative-Stress-Related Genes and Proteins in the Porcine Endometrium—An In Vitro Study
Source: Int J Mol Sci. 2024 Jun 25;25(13):6931. doi: 10.3390/ijms25136931 (PMC11241303; doi:10.3390/ijms25136931)

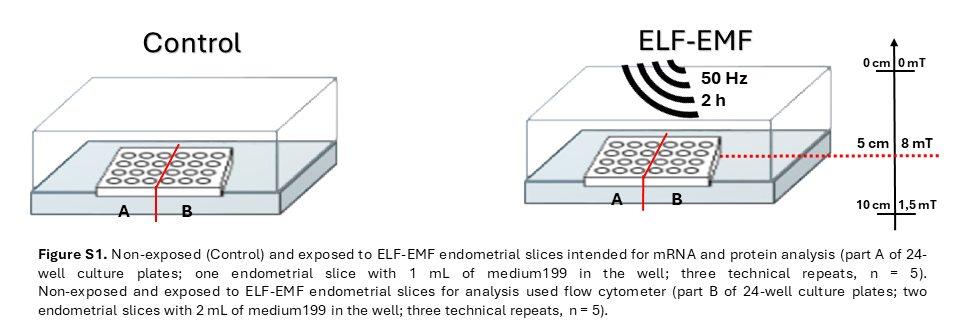

Supplement: Supplementary file 1 [file ijms-25-06931-s001.zip › Figure S1.tif]

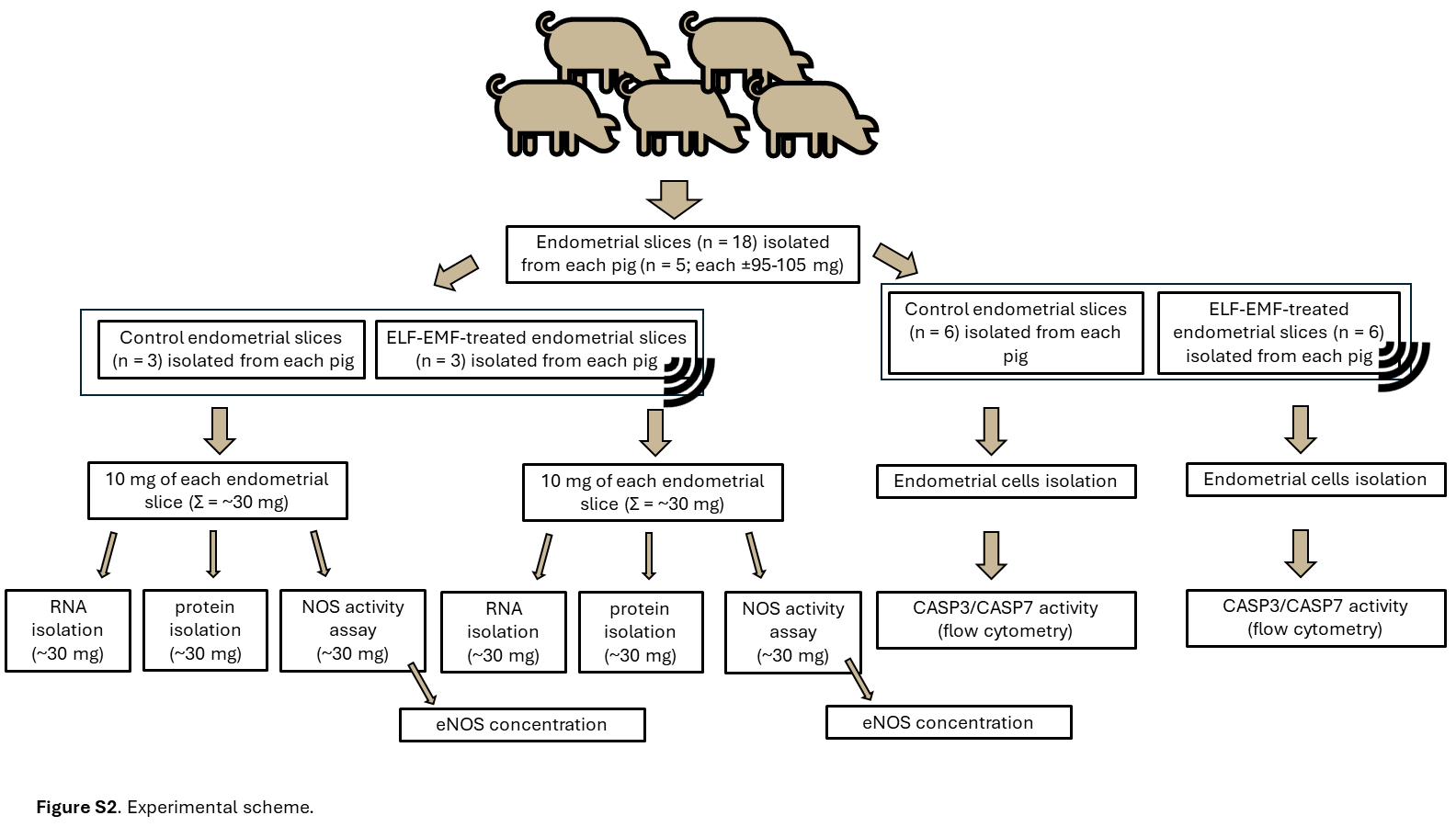

Supplement: Supplementary file 1 [file ijms-25-06931-s001.zip › Figure S2.tif]
